# Implementation of a Distributed Framework for Permissioned Blockchain-Based Secure Automotive Supply Chain Management

**DOI:** 10.3390/s22197367

**Published:** 2022-09-28

**Authors:** Saima Zafar, Syed Faseeh Ul Hassan, AlSharef Mohammad, Ahmad Aziz Al-Ahmadi, Nasim Ullah

**Affiliations:** 1 Department of Electrical Engineering, National University of Computer and Emerging Sciences, Lahore 44000, Pakistan; 2 Department of Electrical Engineering, College of Engineering, TAIF University, Taif 11099, Saudi Arabia

**Keywords:** authentication, automotive supply chain, blockchain, decentralized data register, distributed ledger, Hyperledger Fabric, security

## Abstract

An automotive supply chain includes a range of activities from the concept of the product to its final transfer to a customer and subsequent vehicle maintenance. The three distinct stages of this chain are production, sales, and maintenance. In many countries, automobile records are not available to the public and anyone who has access to the central database or government systems can tamper with these records. In addition, used vehicle maintenance and transfer histories remain unavailable or inaccessible. These issues can be overcome by incorporating state-of-the-art blockchain technology into automotive supply chain management. Blockchain technology uses a chain of blocks for distributed transfer and storage of information, creating a decentralized data register that makes records of any digital asset tamper-proof and transparent. In this paper, we implement a permissioned blockchain-based framework for secure and efficient supply chain management of the automobile industry. We employed Hyperledger Fabric; an enterprise-grade distributed ledger platform for developing solutions. In our solution, the blockchain is customized and private in order to ensure system security. We evaluated our system in terms of memory cost, monetary cost, and speed of execution. Our results demonstrate that only 346 MB of extra memory space is required for storing the automotive data of 1 million users, thus rendering the memory cost negligible. The monetary cost is insignificant as all open source blockchain resources are employed, and the speed of record update is also fast. Our results also show that the decentralization of the automotive supply chain using blockchain can implement system security with minor modifications in the established configuration of the web application database.

## 1. Introduction

**Figure 1:**
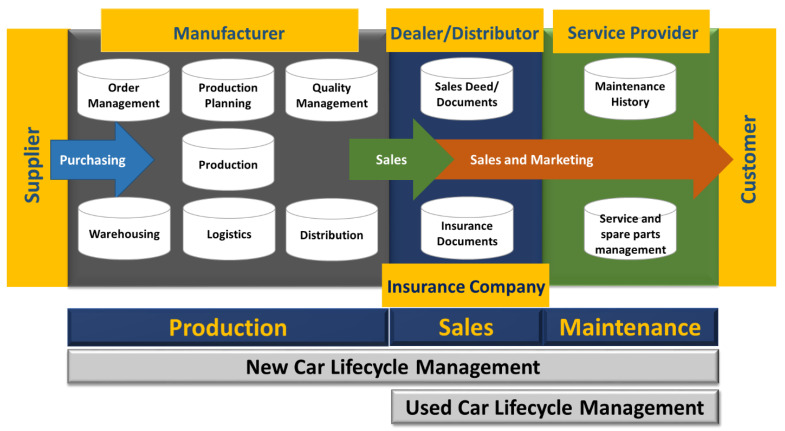
Automotive supply chain processes.

An automotive supply chain is a connection of all stakeholders, assets, industries/businesses, and undertakings involved in the promotion or distribution through which an automobile reaches the customer. It establishes a link between channel affiliates such as suppliers, manufacturers, wholesalers, distributors, retailers, and the customer [1,2]. Supply chain management is a process of organizing and regulating supply chain operations. The key players in the automotive supply chain can be identified on the basis of three explicit stages, which are production, sales, and maintenance. These key players are the manufacturer, dealer, service provider, and customer as shown in Figure 1. In many countries of the world, at the time of purchase, the record of a new or used automobile is preserved in systems comprising centralized databases managed by government authorities. These records are not available to the public, and anyone who has access to the central database or government system can tamper with these records. Due to a lack of records or insecure records, the purchaser of a used vehicle remains unaware of the maintenance and transfer history of the vehicle. This issue is more pronounced in the case of used automobiles. It was reported in a survey conducted in the USA that people prefer to buy used cars rather than new ones due to higher taxes imposed on the purchase of new cars; thus, more than 71 percent of automobile buyers prefer to buy used cars [3].

In order to maintain data and the history of used cars, both local and imported, a company named Carfax Inc. was founded in the year 1984 and soon logged more than a million records of cars in the US and Canada [4]. This practice ensued in many other countries and automobile records became digitized. However, these huge centrally maintained digital records remain highly insecure, susceptible to tampering and hacking, and their integrity remains questionable. Thus, it is imperative to ensure reliable, tamper-proof, and trusted logging and maintenance of automobile records which should be accessible to all involved key players or parties. We believe that the above-mentioned trust and security issues in automobile supply chain management can be resolved by employing state-of-the-art blockchain technology. The primary motivation behind our work is to address the above-mentioned challenges by developing and testing a blockchain-based system using Hyperledger Fabric. The reason for using Hyperledger Fabric instead of Ethereum is two-fold. First, Hyperledger Fabric allows components to be plug-and-play, and its modular design satisfies a broad range of industry use cases. Secondly, it is a limited-access blockchain contrary to Ethereum which is a public network. Blockchain technology, also sometimes called Distributed Ledger Technology (DLT), uses a chain of blocks, which allows distributed transfer and storage of information, creating a decentralized data register. By decentralization and cryptographic hashing, this makes the record of any digital asset tamper-proof and transparent, and ideally suited to implement authentication, confidentiality, and secure data storage [5]. This technology has attained pervasive application in various domains including the pharmaceutical industry, healthcare, agriculture food supply chain, real estate, and banking, among others, to renovate existing applications based on centralized databases into distributed blockchain-based systems to enhance security and performance [6,7,8,9,10,11,12].

**Figure 2:**
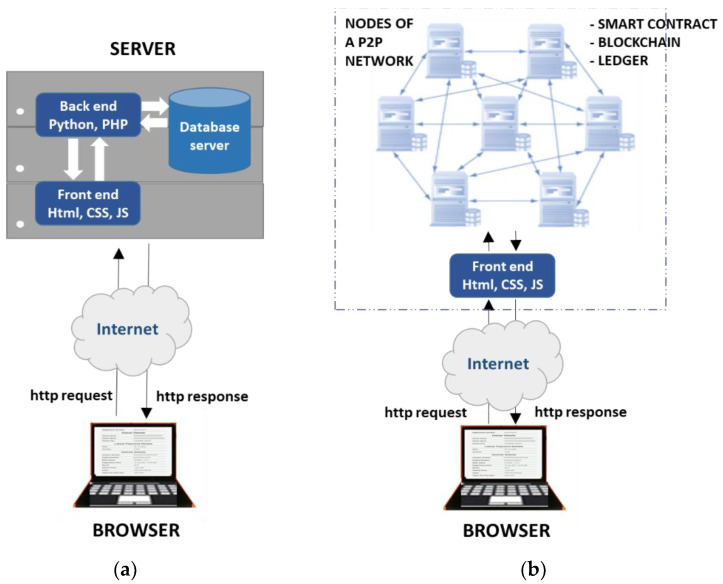
Workflow of (**a**) centralized web application versus (**b**) distributed web application.

In automobile supply chain management, blockchain technology can be implemented for registering and managing decentralized automobile records such that the various parties have access to these records as per their rights and privileges. In case of an attempt to tamper with the chain of records, a consensus algorithm would assist in tracing nodes where a tampering or hacking attempt is made. In this network, the rights to update the chain to the nodes are also based on solving an algorithm. Traditionally, typical web applications communicate with a centralized server in a client-server model as shown in Figure 2a. These applications are generally classified as static or dynamic. The static applications are less interactive and display fixed content or a web page to every user. In a static web application, when a client sends a Hyper Text Transfer Protocol (HTTP) request using a browser for a web page, the server locates the requested web page (in the form of a base HTML file and a number of referenced objects) from its storage and sends it back to the client as an HTTP response. The dynamic web application maintains databases such as sports updates, stock prices, news, weather reports, or some kind of user data. The client can make different queries about data and the server generates a dynamic web page as per the requested data. In dynamic web applications, when the user requests specific data from an application server, the server calls the application program to deal with the request, and the program executes and produces an HTML-based output. Then, the server sends the results back to the client. The browser runs the provided HTML code and displays the result to the user. Decentralized applications (dApps) are a novel paradigm for web applications that are extensively secure. Contrary to typical web applications, dApps have their protocols and data encrypted and stored on a blockchain. dApps have been commercialized by distributed ledger technologies such as the Ethereum Blockchain, where dApps are often mentioned as smart contracts. Blockchain technology ensures data security and integrity by employing decentralization which is reflected by the workflow diagram shown in Figure 2b.

dApps are defined as a software system that uses DLT technology such as a blockchain as a central hub to store and exchange information through smart contracts [13]. The first application of this technology was the invention of the electronic currency Bitcoin in the year 2008 [14]. It was a decentralized system with no office and without any physical appearance, the funds were distributed to all nodes by a decentralized system, and the records of all transactions were shared with all the nodes present in the system. Proof of Work (PoW) is the consensus algorithm used by Bitcoin to make sure that the new block which is being added to the blockchain is safe and secure. This approach also solved issues with previous currencies that failed due to double spending, as they did not have any consensus algorithm. Bitcoin Technology was implemented in 2009 and since then different revisions have been made by data scientists but the main core remains the same, which is to provide contactless, secure, and transparent transactions to all peers in the network. An important aspect of Bitcoin is that there is no owner of this currency. The control of the currency is distributed to all nodes that can vote for any consensus being called by the system to check the validity of a block.

The distributed and decentralized model of blockchain has been inspirational for a number of networking applications that were previously based on the centralized model and employed a single central database and thus lacked security, reliability, efficiency, and, above all, transparency. It is imperative and timely to redesign such applications and systems on the tracks of blockchain to incorporate the above-mentioned features in them. One such indispensable system is automobile supply chain management, and this paper presents the framework and implementation of permissioned blockchain-based system for secure and efficient supply chain management in the automobile industry. We implemented our framework by employing the Hyperledger Fabric; an open-source enterprise-grade distributed ledger platform for developing solutions and applications. In our solution, the blockchain is customized and private to ensure system security. We evaluated our system in terms of memory and monetary costs, and speed, and our results demonstrate that our system is feasible, efficient, and secure compared to the conventional centralized approach. We summarize the main contributions of our work as follows:

1. A permissioned blockchain-based distributed and decentralized framework for the automotive supply chain management.
2. Design and implementation of the given framework using the Hyperledger Fabric.
3. Performance evaluation of the designed system in terms of memory cost, monetary cost, and speed of execution.

The rest of the paper is organized as follows: In Section 2, we present a literature review and summarize notable work in this domain. In Section 3, we explain some of the key concepts that are central to the blockchain model, how a blockchain works, and clarify the difference between a public and permissioned blockchain. Section 4 presents the given framework. Section 5 presents the implementation details of our proposed framework, its performance analysis, and results. Finally, Section 6 concludes the paper.

## 2. Literature Review

Blockchain has found widespread applications in implementing security, reliability, and trust among network entities in a wide spectrum of applications. These include, but are not limited to, healthcare, asset management, insurance and payments, smart appliances, government, passports, personal identifications, certificates, trading, and banking transactions [6,7,8,9,10,11,12]. Our main focus is the automobile industry; therefore, we review notable work in this domain. Blockchain-based decentralized application architecture has been adopted to implement security in a diverse spectrum of the automobile industry, which includes vehicular communication in a smart city, autonomous vehicles, in-vehicle infotainment, Internet of Vehicles (IoV), automobile value chain management, and supply chain management [15]. 

Fraga-Lamas and Fernandez-Carames proposed a blockchain-based trust management system, which is an integrated system to build a decentralized, secure, and reliable trust-based management scheme to evaluate trust among network entities that comprise the connected vehicles in an Intelligent Transportation System (ITS) [16]. The authors state that smart cities are an opportunity to improve the safety and well-being of society, transportation plays an important role in this process, and trust must be built between connected autonomous vehicles to ensure safe transportation. They assert that blockchain technology offers a mathematically verifiable framework for trust building. They proposed a blockchain-based trust management scheme intending to establish a trustworthy environment for connected autonomous vehicles. Sharma et al. proposed a blockchain-based distributed framework for the automotive industry in a smart city including a node selection algorithm, and evaluated their framework using a private Ethereum blockchain platform [17]. Ferdous et al. state that several entities are involved in the lifespan of a smart car including manufacturers, owners, government authorities, and service providers, and there is a need to manage and share data among them in a secure and trustworthy manner to ensure that the data is not manipulated or forged. They propose a blockchain-based architecture to enable the car owner to create an immutable record of data called the autobiography of a car [18]. Kandah et al. consider blockchain technology as a solution for trust management in smart cities and connected vehicles and propose an integrated system for developing distributed, tamper-proof, and dependable trust management [19]. 

Another blockchain-based solution for IoV for smart cities has recently been put forward by Dwivedi et al. who proposed to replace a centralized intelligent transport system with a blockchain-based decentralized vehicular ad-hoc network in order to support data immutability [20]. The authors also proposed an authentication protocol, a consensus mechanism, and a smart contract mechanism and verify their solution by examining its communication, computation, and storage overhead. Chen et al. presented a blockchain-based data trading approach for IoV [21]. They validated their proposal by mathematical analysis. Abbas et al. performed an extensive survey of blockchain-based authentication in IoV [22]. They argue that in IoV, a huge amount of data is exchanged among the different communication entities over highly insecure wireless links which necessitate the use of technologies such as blockchain. They gave a detailed comparative study in terms of techniques, network models, and attacks counteracted. Another similar survey was performed by Wang et al. who analyzed the combination of the blockchain technology and the IoVs from different aspects and highlighted future directions for this integration [23]. Blockchain has been implemented to secure data exchange in Autonomous Vehicles (AVs). A notable study in this domain was presented by Hasan et al. [24]. The authors state that the ascent of AVs demonstrates that these are going to be the juggernaut in the vehicle industry. The innovation utilized by the AVs makes ride-sharing an unmistakable and adaptable route for transportation. For this situation, clients who are sharing the AV, and the AV itself, ought to be imaged to check one another. When a client demands a ride offer or lease, the reacting AV gadget does not have the adequate capacity for storage, processing, or confirmation. In the current security arrangements, each AV gadget regularly depends on a confided outsider, which raises trust issues. If the trusted outsider is an insurgent or loses validity, the entire framework would crash. Hasan et al. [24] suggest incorporating blockchain to produce a safe and dependable way to impart trust among gadgets. Zielinska et al. employed the concept of a blockchain technology model with respect to the charging process of electric vehicles [25]. Their model supports partial and complete decentralization of the procedure without road units and without supervision. Another noteworthy application of blockchain is secure communication and exchange of information among Electronic Control Units (ECUs) in vehicles as investigated by Alam et al. who propose the use of symmetric key cryptography and elliptic curve-based Public Key Encryption (PKE) for implementing confidentiality and the use of digital signature for integrity and authentication [26]. The authors introduce a blockchain-inspired mechanism for securing the ECU data.

The use of blockchain for secure and reliable storage and management of automobile data is directly related work to our research, and we highlight some notable work in this domain. Brousmiche et al. emphasized the need for the management of automobile data over its life cycle and the efficacy of securing it with blockchain technology to prevent vehicle fraud with the involvement of all stakeholders [27]. The authors presented a hybrid cryptographic protocol to warrant access to vehicle data to the concerned stakeholders. A closely related work is by Masoud et al. who presented a novel public blockchain-based used motor vehicle history reporting system named CarChain [28]. Instead of constructing their framework based on existing popular public blockchain networks, their framework builds a peer-to-peer (P2P) overlay network that broadcasts transactions. Using this framework, the car owners, repair companies, and insurance agencies register and add car histories in a hyperledger that has the potential to store information along the production chain to allow for association and warrant information integrity. Similar work was presented by Jiang and Sun that secures vehicle condition data of used vehicles [29]. Another closely related work is by Reimers et al. who presented a proof-of-concept prototype of the integration of blockchain and the Internet of Things (IoT) in a car supply chain [30]. The authors state that with distributed ledger technologies such as blockchain, a collaboration system can be formed, offering better services than a traceability system because in addition to speedy product traceability it offers a refinement of cross-organizational business processes and distribution of information to the end customer. The authors emphasized the importance of secure and reliable integrated information created in the pre-sales and post-sales processes of a product life cycle. Contrary to our work, their work involved IoT-based data collection through sensors and the use of Message Queuing Telemetry Transport (MQTT) protocol instead of HTTP. Some interesting works have reported on using blockchain for secure car registration, such as [31,32,33], vehicle identity verification [34], and vehicle authentication [35]. These studies focus on one aspect of the vehicle lifecycle, while our study is a comprehensive framework covering the complete vehicle lifecycle. 

Another work related to the use of blockchain for securing IoT-based applications is by Singh et al. who called blockchain a game changer for securing IoT data and provided a model for the security of IoT using blockchain [35]. Insurance is an essential component of the automobile life cycle. Wan et al. incorporated blockchain-based security and other features into the usage-based insurance of automobiles [36]. In such systems, the insurance premiums are decided according to the usage and driving pattern of automobiles such that the insurance costs are reduced for safe drivers. Currently, these systems depend on centralized insurance companies as intermediaries to manage insurance, which is costly in terms of time and money. The authors presented a privacy-preserving and decentralized scheme using the blockchain to record encrypted driving data, and a smart contract executing on the blockchain to compute insurance premiums. We conclude that the notable recent work in the use of blockchain for the automobile supply chain, which includes protocols to control sharing and access to vehicle data, systems to access vehicle data, and IoT-based data collection systems, overlook post-sales information update and maintenance, and mostly employ empirical analysis or overlay network to implement and analyze their systems. They do not address the need for a comprehensive framework over the complete vehicle lifecycle and its verification by employing the most suitable, popular, and contemporary Hyperledger Fabric technology for blockchain implementation. 

## 3. Materials and Methods

A blockchain is an expanding record of entries, called blocks, connected using cryptography. Each block comprises a cryptographic hash of the previous block, a timestamp, and transaction data (usually represented as a Merkle tree). It is a distributed ledger, like a database, which is decentralized, distributed, encrypted, and unalterable. Decentralized means there is no involvement of a third party, distributed means it is spread across a peer-to-peer network, encrypted means the data is protected using cryptography, and unalterable means that once data are added to the ledger, they cannot be removed or altered. In short, a blockchain is a ledger through which data are added and updated in real-time via the consensus of the nodes running the software in the network. In a peer-to-peer network, there is no central entity, all nodes in the network are peers and function as validators of the state of the ledger. The central processes of blockchain technology exploit state-of-the-art cryptography, which is used to build the blocks.

### 3.1. How Blockchain Works 

**Figure 3:**
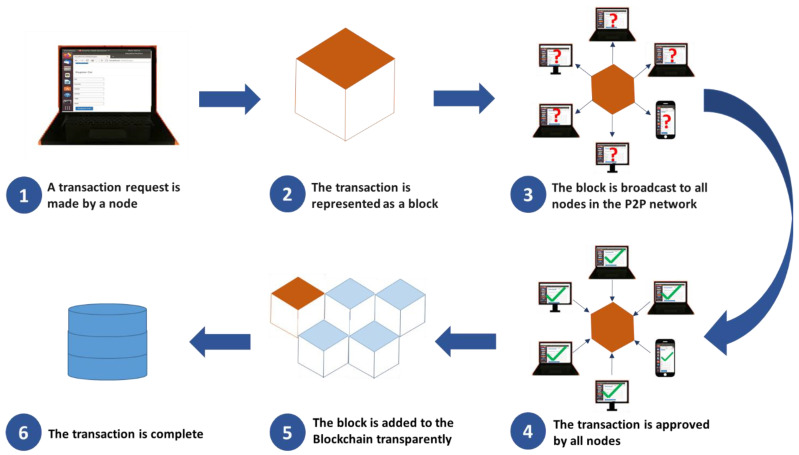
How blockchain works.

The working of blockchain is shown in Figure 3. The nodes are connected to a P2P network and each node has a full copy of the blockchain. When a transaction is made, a new block is created which must be verified before it is added to the chain. This verification is called consensus, which is achieved by a consensus algorithm that runs at the network nodes. If more than 50% of the network nodes verify a transaction, the block is added to the blockchain. The records in a block depend on the type of the application; for example, in healthcare applications, the data in a block are the medical records of patients, while in the bitcoin application the records are the transaction details. Since a block also contains a hash of the preceding block; thus, it produces a chain of blocks called a ledger. The chain of blocks protects the data in a block against tampering because modification of data in any one block in the chain would also change its hash, which makes all of the succeeding blocks invalid as they would not have a valid hash of the preceding block. The distributed nature of blockchain makes the data in the blockchain tamper-resistant. It becomes almost impossible to modify data once it is recorded inside a blockchain. In order to modify data, an attacker has to change all blocks in the chain and for that, he must have control of over 50% of the network.

### 3.2. Proposed Distributed Framework for Automotive Supply Chain Management

**Figure 4:**
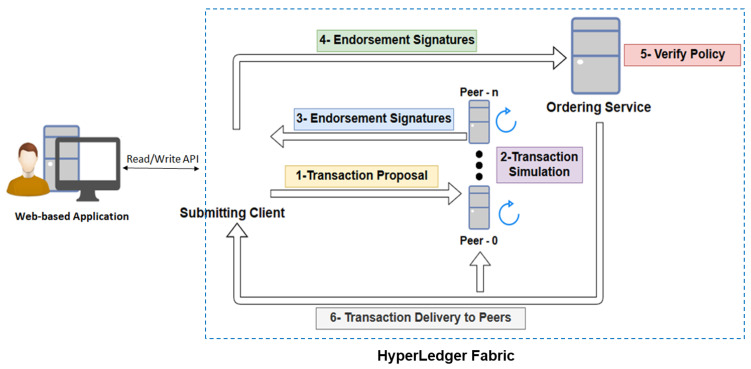
The structure and flow of the proposed distributed framework for automotive supply chain management.

The structure and flow of our proposed framework for a permissioned, blockchain-based, secure automotive supply chain is shown in Figure 4. Since our system is concerned with integrity protection and trust achievement for a web-based application, it is important to implement our solution over a functioning web application. The application has the following functionalities:

- It incorporates authorization to the various users based on their privileges.
- It allows permissioned users to insert, update and delete data,
- It allows multiple data owners to store data.
- It provides an interface for the decision-maker, to view the uploaded data.

**Figure 5:**
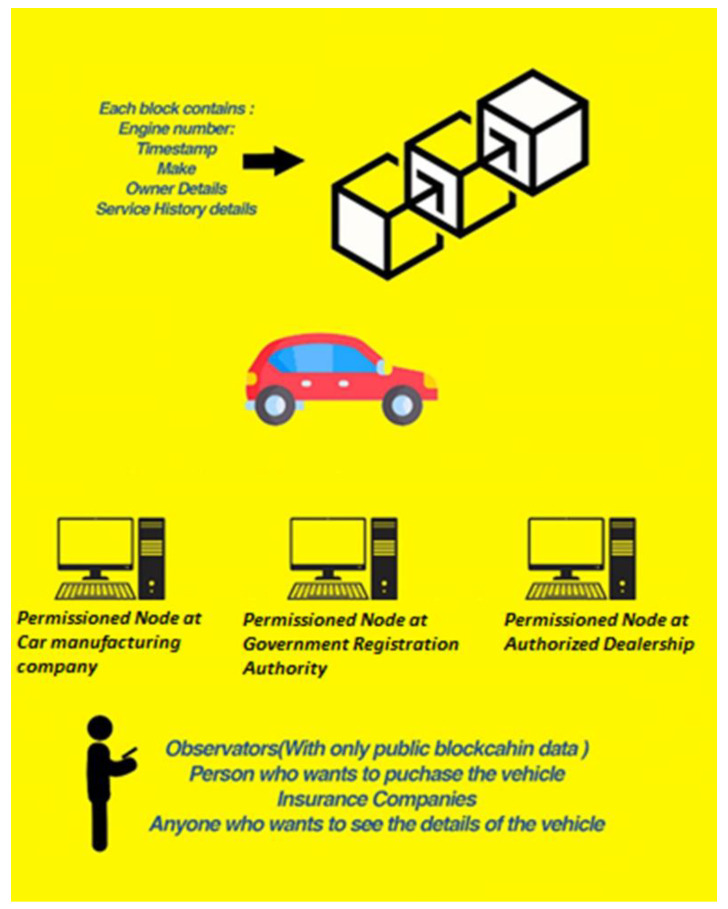
Permissioned nodes in the network.

The blockchain-based implementation in our framework exploits a small-scale implementation of a Hyperledger Fabric network as a data storage point (in place of a centralized database). To simulate the distributed structure of an actual Hyperledger Fabric network, the nodes/peers run in separate container/docker environments. The peers have the smart contract/chaincode installed on them, which are the software instructions used to read and update data on the blockchain ledger. The chaincode is written in the Node.js programming language. We made a unique wallet on Node.js and then started Hyperledger Fabric after registering the users who could perform tasks based on privileges set by the admin. Figure 5 shows the entities with permission to enter vehicle records in our framework which are as follows:

- Car companies.
- A Government car registration authority.
- An authorized dealership to update the service history of a car.

They all have the permission to add data to the blockchain but through a consensus algorithm that makes sure that the entered data matches the previous hash so that invalid data are not added to the blockchain. The observatory entries can be those with limited access to the blockchain such as the customer who wants to see the details of their data in the government database, or a car buyer who wants to see the ownership of a used car along with its service history. This system helps to secure car data along with its history. A permissioned user has full access to the system database and, can insert data into the decentralized database and can update, modify or delete it. The observer can register themselves and can enter/edit some details such as the insurance company or a car’s buyer, who can log in and check the details of a car.

The result of the execution of a chaincode is success or failure. The chaincode does the following:

- It verifies that the transaction request is from a privileged user.
- It supports the nodes to implement and validate transactions at the network.

Upon receiving a read/write request by the application user, the read/write API queries or invokes chaincode on Hyperledger Fabric. The transaction requests are forwarded to the Hyperledger Fabric network, where they are endorsed and validated. The response of validation is sent to the user. Upon an invocation request, the transactional flow of the Hyperledger Fabric consists of six steps as shown in Figure 4. These steps are explained as follows:

1. The invocation request sends the transaction proposal to the endorsing peers.
2. The endorsing peers simulate the transaction proposal over the copy of the ledger that they have. The peers then create a read/write set, i.e., what is read from the ledger and what would be written on the ledger while simulating the transaction proposal on the current state of the ledger.
3. After cryptographically signing, peers send the results, i.e., read/write set back to the client.
4. The client then sends the signed and endorsed transaction along with read/write set to the ordering service.
5. The ordering service consists of a cluster of orderer peers. It accepts endorsed transactions to verify its signatures along with policy or chain code. The ordering service then uses algorithms such as Kafka or Solo to produce an order in which these transactions will be added to the ledger. The ordering service then sends the data to committers. The committing peers verify that the read/write set from different endorsing peers matches the current state of the ledger.

If the match is successful, the committers write the transaction to their ledger and inform the client about it.

**Figure 6:**
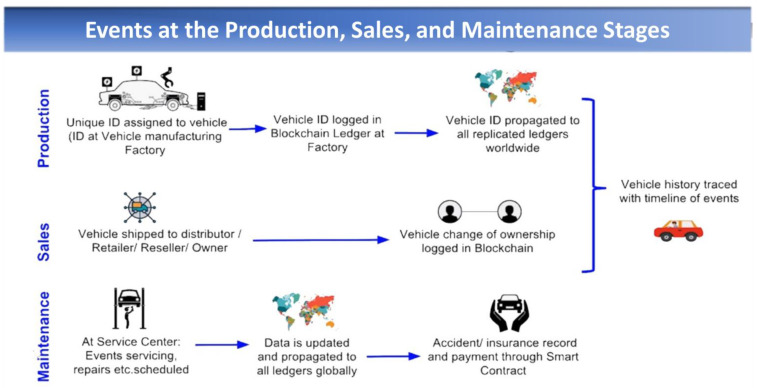
Events (to be saved in the blockchain) at the various stages of the automotive lifecycle.

Our framework covers three stages/phases of the automotive supply chain, namely production, sales, and maintenance, as explained in Figure 6. Each vehicle is assigned a unique ID at the time of manufacture or import (in the case of a used vehicle). This ID is logged in the blockchain ledger and is propagated to all replicated ledgers worldwide. Whenever the vehicle is shipped to the distributor/dealer or to the customer, a change of ownership is logged in the blockchain. In the post-sale scenario, all maintenance activities of vehicles at the service center are logged in the blockchain. Insurance records are also updated accordingly. The users or subscribers of the system are car manufacturers, car dealers, car owners, service centers, and insurance agencies. The system operates as per smart contract between all involved parties. These contracts work similarly to Ethereum, and save the meta-data of the saved information [37]. The system also generates and adds transaction blocks to the chain every time a transaction is carried out. The responsibility to update the records lies with the production, sales, and services organizations. These rights and privileges are coded as part of the smart contract implemented via chaincode, as discussed in previously.

## 4. Implementation Results

**Figure 7:**
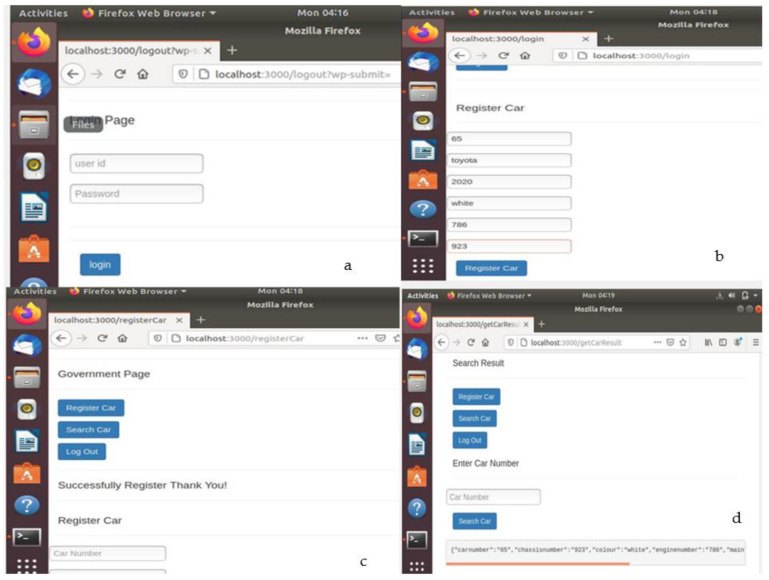
(**a**) User login. (**b**) Car registration interface. (**c**) Successful registration. (**d**) Blockchain successfully created.

**Figure 8:**
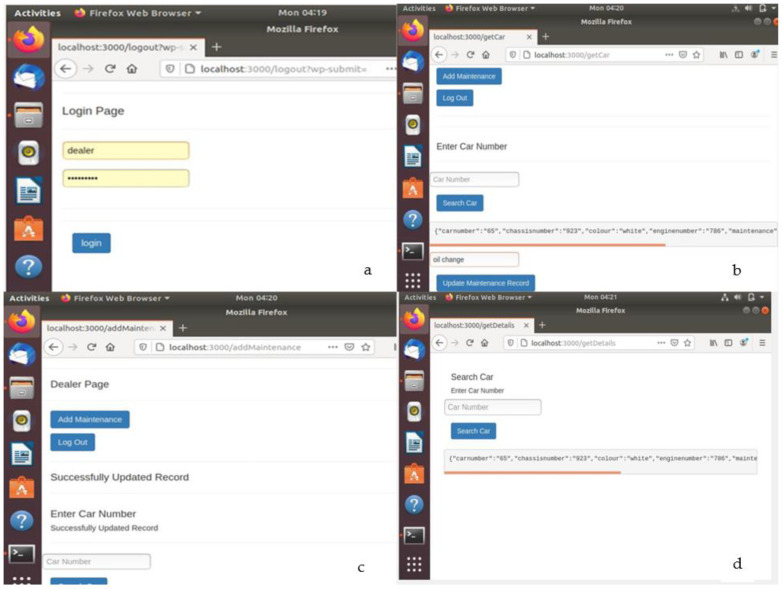
(**a**) Dealer login. (**b**) Updating car maintenance record in blockchain. (**c**) Successful record update. (**d**) Search for car history including maintenance record.

**Figure 9:**
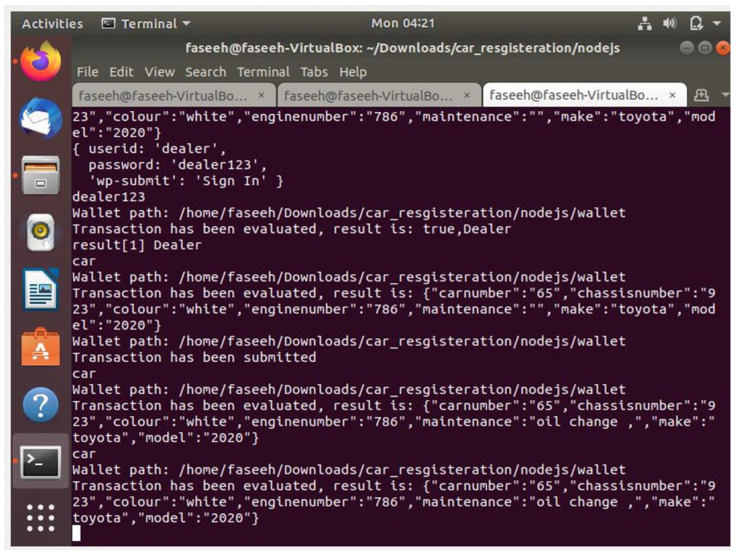
Blockchain backend updates.

Figure 7 shows web pages and activities pertaining to the successful creation of car records in the blockchain by any one of the permissioned users. Figure 7a shows the login page for the permissioned user who can log in using a valid User ID and password. Figure 7b shows the interface which appears when after successful login, a permissioned user presses the ‘Register car’ tab and enters car record data. When valid car data are entered into the various fields and saved, a new block is created and appended to the blockchain. Figure 7c shows the successful registration of a car, and Figure 7d shows the car records displayed when the user searches for a car record using its registration number. Figure 8 shows the web pages and activities pertaining to an authorized dealership that is permissioned to update maintenance records against a registered car. After successful login, the dealer searches for a registered car by entering its registration number and then updates its maintenance record. At this time, a new block is created and appended to the blockchain. Upon search by a user, the updated records are displayed as shown in Figure 8d. Figure 9 shows the backend updates to the blockchain as a result of activities performed using the frontend webpages.

Qualitatively, the benefits of using a permissioned blockchain-based decentralized system as opposed to the centralized database-based approach are as follows:

1. Access control: Only permissioned users have access to configure/alter the blockchain.
2. Transparency: The records are transparent. They are replicated thus every participant in the chain has the same copy of the ledger.
3. Encryption: The transactions are encrypted, which means data at all nodes is encrypted, only accessible to the permissioned users.
4. Immutability: Once a block is added to the chain, it is immutable which means it cannot be tampered with or deleted. A new block can be added and updated but it always remains connected to its previous block. For example, the number of owners a car has had cannot be altered or denied.
5. Undisputed: Since blockchain is a shared ledger with a copy available at every node, and it cannot be modified without consensus therefore there cannot be any dispute about the ledger.
6. Ingenuity: Above mentioned attributes result in lesser disputes, and thus fewer resources expended to resolve disputes.

To quantitatively analyze our implemented framework, we evaluated the memory cost of the system and the speed of execution. Our analysis is explained below:

### 4.1. Memory Cost Analysis

Our proposed framework can be implemented in any country’s vehicle tracking system developed by its government. Generally, the required information to be stored and accessed remains the same. We designed our records based on the information stored in the government of Pakistan’s Punjab excise and taxation department’s vehicle verification system. This information includes vehicle details such as car ID, chassis number, color, engine number, maintenance record, make name and model. More information can be added if required, such as vehicle price, registration date, owner details, service history, etc. Our proposed solution requires very few changes to the existing schema of a web application database. Each data owner has a public/private key pair for digital signatures.

Thus, we need a collection/table to store the public keys of respective data owners in the database. Table 1 shows the “public_key” collection (‘Table’ in SQL) to be added to the database to store the public key of the data owner. The “public_key” collection has two fields “userID” and “public key”. The “userID” stores the unique identification string and the “public key” stores the public key of the car.

**Figure 10:**
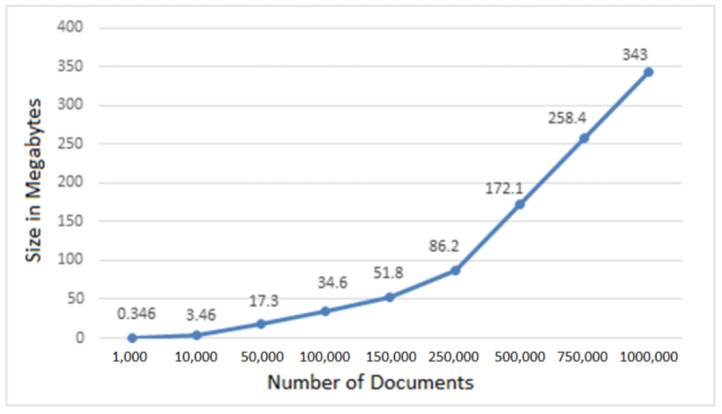
Number of documents versus data-size.

Using “db.collection.stats()”, we can obtain the size of a document in a mongo collection. For each document (‘row’ in SQL) in the “public_key” collection, it takes 0.346 KB or 0.000346 MB of extra space in memory. Therefore for 1 million users, it would take 346 MB of extra space in existing web applications. Figure 10 shows the relationship between the number of documents in the ‘public_key’ collection and memory size in MB.

### 4.2. Monetary Cost Analysis

The system has a very low cost, as most of the resources of blockchain are open source. We used free open-source software to convert data which are already available and stored in the cloud or on a computer. Therefore, the monetary cost for this project is minimal if it is applied to the vehicle tracking records or automobile supply chain records of a country.

### 4.3. Record Update

**Figure 11:**
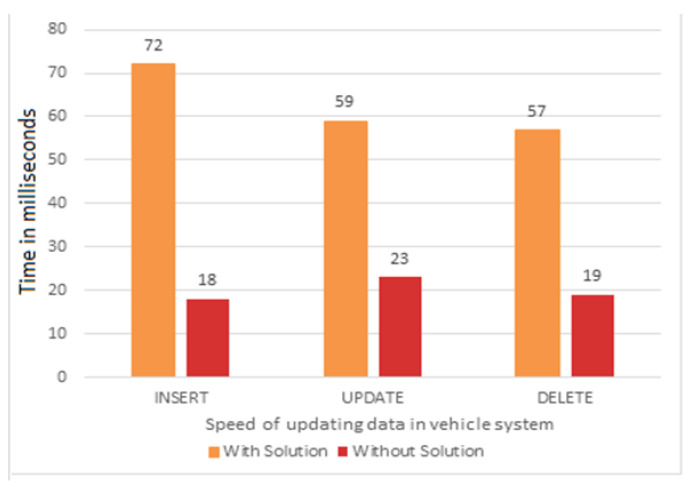
Execution speed of records update.

To compare the speed of records update of our framework with a case in which a centralized database is employed instead of a Hyperledger Fabric network, we used MongoDB. The speed of record update is recorded both when the centralized database is used and when the Hyperledger Fabric network is used [38]. Our results show that the use of a decentralized database results in reduced delay in record updates compared to a centralized database. The requests for car registration record entry (Insert), car data record update (Update), and car data record deletion (Delete) were invoked multiple times. The speed of execution comparison between the centralized and the decentralized system is shown in Figure 11, which clearly shows the advantage of fast record updates in the case of the blockchain-based system. Although it seems paradoxical, as the Hyperledger Fabric network is expected to cause more delay due to the execution of chaincodes, the results are opposite because of a low number of endorsing peers and simple chaincodes used in our implementation, while MongoDB is slow in the absence of indexes. The speed of execution of the Hyperledger Fabric network can be further improved by various techniques; therefore, in the future, it is expected not to be a limitation at all [39,40].

## 5. Discussion

We studied the application of permissioned blockchain to the automotive supply chain using Hyperledger Fabric technology. The permissioned blockchain is a promising technology with the potential to secure and automate supply chains. We explain the difference between the public and the permissioned blockchain as follows. Public blockchains are open to anyone, which means anyone can join the network without permission [14]. Anyone can make transactions, can read transactions, and can take part in consensus. To become a part of the network, one needs to download the open-source code and run it on a local machine. In public blockchains, the participants have anonymous identities, using which they can make transactions, and take part in a consensus mechanism i.e., the process of validating blocks of transaction and adding them to the chain. Therefore, in a public Blockchain, anybody can make valid transactions that are added to the chain. A public Blockchain uses PoW and Proof of Stake (PoS) as consensus algorithms. Bitcoin and Ethereum are examples of public blockchains [14,37].

In Bitcoin, it takes 10 min for a transaction to become a part of the chain. Permissioned blockchains maintain the identity of participants and regulate the role of participants, which provides more trackability and efficiency. Thus, with a permissioned blockchain deployed in an organization, the organization controls the read or write access to the blockchain. As participants do not have anonymous identities, it helps in better auditing and trust. Permissioned blockchains are faster than public blockchains, as they do not need consensus algorithms. Our implementation results analyzed the memory and monetary costs of implementing the decentralized permissioned blockchain-based supply chain, and the results show that both costs are acceptable and do not pose a big challenge to this implementation. Our proposed solution mandates minimal changes in the existing structure of a web application database. The monetary cost is manageable, and the speed of record updates is drastically improved due to the decentralized nature of the database and automated authentication and record updates. This research is expected to encourage additional research endeavors for the effective wide-scale adoption and implementation of permissioned blockchain as a promising technology for automated automotive supply chain systems. In the future, we plan to extend our framework to include the automotive manufacturing value chain. Blockchain has the potential to revolutionize supply chains due to security and transparency. It is the need of the hour to extend blockchain applications to make businesses take advantage of this technology.

## 6. Conclusions

The rising demands for automated supply chain systems have endorsed the need for automation of vehicle supply chain and lifecycle management across the world. However, most records are saved in centralized databases that remain vulnerable to tampering and forging by the involved parties at any stage of the chain, resulting in a lack of trust. There is a need for a secure and tamper-proof digital automotive supply chain management system, and blockchain, with features such as immutability and decentralization, is a suitable technology to be implemented in such systems. There are different types of blockchain, public and permissioned, and various platforms to implement blockchain frameworks. In this paper, we present a permissioned blockchain-based framework for the automotive supply chain and implement it in Hyperledger Fabric. We believe that permissioned blockchain with its access control layer is the most suitable type to be implemented for the automotive supply chain, and Hyperledger Fabric which is an enterprise-grade permissioned distributed ledger framework for developing solutions and applications, is the most suitable platform due to its modular and versatile design. The platform allows entities with different roles. We implemented two roles: permissioned nodes which are car manufacturing companies, government authorities, and authorized dealerships; and observers which can be car buyers who can view the trusted information for decision-making. Our implemented system is efficient in terms of memory and monetary costs, and speed of execution. We conclude by accentuating the fact that secure automated supply chain systems are inevitable in the future digital era, and permissioned Blockchain technology will find a key place in implementing security and trust in these systems.

## Figures and Tables

**Table 1:** Public key collection in database.

Collections	Fields in Documents
Public_key	userID, publickey

## Data Availability

Not applicable.
